# Bone Mineral Density, Osteoporosis, and Fracture Risk in Adult Patients with Psoriasis or Psoriatic Arthritis: A Systematic Review and Meta-Analysis of Observational Studies

**DOI:** 10.3390/jcm9113712

**Published:** 2020-11-19

**Authors:** Tai-Li Chen, Jing-Wun Lu, Yu-Wen Huang, Jen-Hung Wang, Kuei-Ying Su

**Affiliations:** 1 Department of Medical Education, Hualien Tzu Chi Hospital, Buddhist Tzu Chi Medical Foundation, Hualien 970, Taiwan; terrychen.a@gmail.com (T.-L.C.); jingwunlu@gmail.com (J.-W.L.); 2 Department of Medical Education, Taipei Tzu Chi Hospital, Buddhist Tzu Chi Medical Foundation, New Taipei City 231, Taiwan; 102311135@gms.tcu.edu.tw; 3 Department of Medical Research, Buddhist Tzu Chi General Hospital, Hualien 970, Taiwan; jenhungwang2011@gmail.com; 4 Division of Rheumatology and Immunology, Hualien Tzu Chi Hospital, Buddhist Tzu Chi Medical Foundation, Hualien 970, Taiwan; 5 School of Medicine, Tzu Chi University, Hualien 970, Taiwan

**Keywords:** bone mineral density, osteoporosis, fracture, bone fragility, psoriasis, psoriatic arthritis, meta-analysis

## Abstract

Introduction: Awareness of psoriasis-related comorbidities has been established in the current guidelines; however, evidence regarding the association of bone density or bone fragility with psoriatic disease remains inconclusive. Methods: We conducted a systematic review and meta-analysis to assess bone mineral density and the risk of osteoporosis and fractures in patients with psoriatic disease, including those with cutaneous psoriasis and psoriatic arthritis. We searched electronic databases for published observational studies. A meta-analysis was performed using the random-effect model. Pooled estimates and their confidence intervals (CIs) were calculated. Small-study effects were examined using the Doi plot and Luis Furuya–Kanamori index. Results: The analysis of the standardized mean difference in the absolute value of bone mineral density at different measuring sites (lumbar spine, femoral neck, and total hip) revealed no significant difference between patients with psoriatic disease and non-psoriatic controls. The pooled results of the adjusted odds ratios (ORs) demonstrated no increased risk of osteoporosis in patients with psoriatic disease. Notably, patients with psoriatic disease had a higher OR of developing bone fractures (adjusted OR: 1.09; 95% CI: 1.06 to 1.12; I^2^: 0%). Conclusion: Patients with psoriatic disease may be more likely to develop fractures compared with non-psoriatic controls. This higher risk for fracture may not necessarily be associated with lower bone mineral density nor a higher risk for osteoporosis.

## 1. Introduction

Cutaneous psoriasis and psoriatic arthritis (PsA) are chronic inflammatory disorders recognized on the spectrum of psoriatic disease [1,2,3]. Genetic and immunologic similarities identified in both the affected skin and joints implicate shared mechanisms in psoriatic disease [4,5,6,7]. Therefore, it is reasonable to consider combining the management of patients with cutaneous psoriasis and PsA in clinical practice due to the common pathophysiological process [8,9]. With an improvement in molecular biology and immunopathology, abnormal bone remodeling discovered in experimental and clinical research has prompted attention toward bone health in psoriatic disease [10,11,12]. Although the current guidelines on psoriatic comorbidities do not include bone health [13] recent studies have indicated that patients with psoriatic disease may be at an increased risk of osteoporosis and fractures [14]. However, the evidence regarding the association of psoriatic disease and bone fragility remains inconclusive.

Reduction in bone mineral density (BMD) in patients with psoriatic disease has been reported in a previous systematic review [15]. The prevalence of osteoporosis in patients with psoriatic disease was reported to be 1.4–68.8% in some studies [15,16,17]. Moreover, patients with psoriatic disease were shown to have a higher risk of developing bone fractures [18,19]. In contrast, other studies reported negative results. Harrison et al., and Busquets et al., revealed no apparent associations between low BMD and psoriatic disease upon clinical observation [20,21]. In several studies, no increased risk of fractures was observed in patients with psoriatic disease than in non-psoriatic controls [22,23]. A complicated and unclear mechanism of bone quality and bone fragility in psoriatic disease may contribute to the controversy above. Additionally, bone fragility and bone strength may be considered beyond bone density alone, making the situation far more complicated [24].

Since the study population, sample sizes, and study designs in individual studies were heterogenous, a comprehensive literature review with meta-analysis is warranted to yield the overall effects. Therefore, in the present study we aimed to perform a systematic review and meta-analysis of observational studies to determine the BMD and fracture risk in adult patients with psoriatic disease.

## 2. Materials and Methods

We conducted this systematic review and meta-analysis in accordance with the Preferred Reporting Items for Systematic Reviews and Meta-Analyses (PRISMA) statement [25] and the Meta-Analyses of Observational Studies in Epidemiology (MOOSE) guidelines [26]. We registered our protocol at INPLASY.COM(registration number: INPLASY202080106). Two investigators (TL Chen and JW Lu) independently searched for articles, collated data, and evaluated the quality of the qualifying studies. In cases of discrepancies between the investigators, a third author (YW Huang) was consulted to reach a consensus.

### 2.1. Literature Investigation and Search Strategy

We searched electronic databases (PubMed, Embase, Cochrane Library, and Web of Science) and Chinese medical databases (Airiti Library and Chinese National Knowledge Infrastructure databases) systemically for studies published from the inception of the relevant database until 15 September 2020. In brief, we used the following terms: “psoriasis”, “psoriatic arthritis”, “bone mineral density”, “osteoporosis”, and “fracture”. The search strategies were modified for the requirements of individual databases, and the details are described in Methods in the Supplementary Materials. Studies in languages other than English or Chinese were excluded. Furthermore, we supplemented our search by examining the reference lists or bibliographies of the available review articles and relevant meta-analyses for additional candidates.

### 2.2. Study Selection and Eligibility Criteria

Peer-reviewed scientific articles were considered for inclusion. Studies in preprint status and those published in open access journals that were absent on the Directory of Open Access Journals (DOAJ) were considered non-peer-reviewed articles and, thus, were excluded. Studies that fulfilled the following criteria were included: (1) those with observational study design (cross-sectional, case-control, or cohort studies); (2) those in which target participants were adults diagnosed with psoriatic disease (cutaneous psoriasis or PsA) based on clinical or histological information; (3) those in which comparison groups included adult controls without psoriatic disease; (4) those in which the outcomes comprised the absolute value of BMD and/or the effect estimates of osteoporosis or fractures; and (5) those in which BMD was assessed at lower extremities (e.g., lumbar spine, femoral neck, etc.) using dual-energy X-ray absorptiometry (DXA), ultrasound bone density measurements, or other effective methods. Case reports, case series, review articles, and abstracts from conference proceedings were excluded. We also excluded animal studies or studies performed in laboratory settings.

### 2.3. Data Extraction and Outcome of Interest

We extracted data regarding the following items: first author, publication year, study design, geographical location, study population (cutaneous psoriasis, PsA, or both), sample size, patient characteristics (age, sex, and body mass index), characteristics of psoriatic disease (disease duration and the usage of potential drugs that may affect bone formation), BMD measurements (device, site, and outcomes), and reported outcomes of osteoporosis and fractures. Systemic corticosteroids, methotrexate, and anti-tumor necrosis factor (TNF)-α agents were considered potential drugs that could be related to bone quality and fragility [27,28,29]. The conflict of interest study was also listed for each study. The primary endpoint was the absolute value of BMD. The secondary endpoints included effect estimates regarding osteoporosis and fractures.

### 2.4. Qualitative Systematic Review

A modified Newcastle–Ottawa Scale (NOS) for non-randomized studies was utilized for methodological quality appraisal of the included studies [30,31]; it consists of the following three domains: the selection of study groups, comparability of study groups, and ascertainment of the outcome of interest. Modified NOS for observational studies were demonstrated in Tables S1–S3 in the Supplementary Materials.

### 2.5. Data Synthesis and Statistical Analysis

Considering the heterogeneity of the study populations, we calculated the pooled estimates and their confidence intervals (CIs) using the DerSimonian and Laird random-effects model [32]. For continuous outcomes (absolute value of BMD), we calculated the standardized mean differences (SMDs) and 95% CIs. SMD was considered because different manufactural modalities were used across studies. For dichotomous outcomes (risk estimates of osteoporosis and fractures), we calculated estimated odds ratios (ORs) and 95% CIs. We focused mainly on the pooled results using maximally-adjusted estimates [33]. However, we still demonstrate the unadjusted ORs for emphasizing the influence of confounding bias [34]. Furthermore, if the enrolled study number of each outcome was less than ten and the pooled effect was statistically significant, modified Hartung–Knapp/Sidik–Jonkman (HKSJ) adjustment was applied to control type I errors and avoid inaccurate CIs [35,36,37]. We contacted the authors for the desired effect estimates and relevant information for studies that did not report the data available for pooling.

Between-study heterogeneity was quantified using the I^2^ statistics [38]. An I^2^ value ≥50% represents substantial heterogeneity. To explore the potential sources of heterogeneity apart from random error, we conducted several predefined subgroup analyses according to the site of BMD measurement, study population, study design, geographic location, age of participants, body mass index (BMI), disease duration, potential osteoporotic/anti-osteoporotic drugs use, and study quality according to NOS.

We also performed a sensitivity analysis to evaluate the influence of each study on the overall effect by omitting them individually. All statistical tests were two-sided, and *p*-value < 0.05 was considered statistically significant. The present meta-analysis was performed using Stata v16 (StataCorp, College Station, TX, USA).

### 2.6. Small-Study Effects

Potential small-study effects, such as publication bias, were examined using Doi plots, a recently developed graphical and alternative method [39]. It has been demonstrated to improve visualized asymmetry with treatment effects on the *x*-axis and a normal rank-based Z-score on the *y*-axis. Doi plot asymmetry was quantified using the Luis Furuya–Kanamori (LFK) index, based on the rank-based measure of precision (Z-score) instead of the standard error in funnel plots [39]. LFK indices less than ±1, greater than ±1 but less than ±2, or greater than ±2 were considered to represent no, minor, or major asymmetry, respectively [39]. Moreover, the LFK index has been demonstrated to outperform Egger’s regression test for possible small-study effects, especially when the study number is small. We applied the Doi plot and LFK index to detect potential small-study effects in several outcomes of interest, which may be ignored by the inapplicability of funnel plots and quantitative approaches, such as the Egger’s *p* test. MetaXL v5.3 (EpiGear International Pty Ltd., Sunrise Beach, Queensland, Australia) was used to generate the Doi plots and calculate the LFK indices [39].

## 3. Results

### 3.1. Search Results

**Figure 1:**
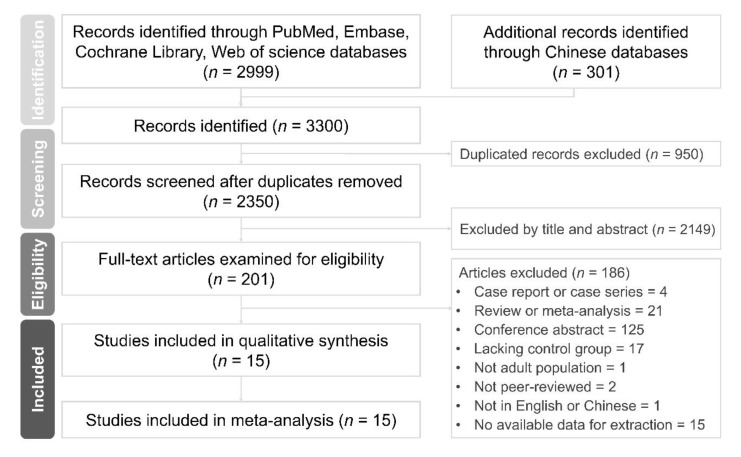
Preferred Reporting Items for Systematic Reviews and Meta-Analyses (PRISMA) flow diagram of the process of screening and including the studies.

The selection and detailed identification processes are summarized in Figure 1. A total of 3300 unique publications fulfilled the initial screening. We removed 950 duplicates, and the titles and abstracts of the remaining studies were screened for inclusion. The full text of 201 studies was retrieved; of them, 15 met the inclusion criteria. Ultimately, 15 observational studies were included in this quantitative meta-analysis.

### 3.2. Characteristics of Qualifying Studies

Table S4 outlines the characteristics of the 15 observational studies [16,18,19,22,23,40,41,42,43,44,45,46,47,48,49]. A total of 1,277,673 participants, investigated between 2009 and 2020, were evaluated. The demographic data and the reported outcomes of interest were summarized. Female-predominant sex distribution could be observed in most of the studies. The participants were mostly categorized as overweight (25 ≤ BMI < 29.9) or obese (BMI ≥ 30) [50].

After critical appraisal of the studies, eight studies were judged to have “high quality” because they scored ≥7 points on the NOS. Additionally, seven articles were deemed to have “moderate quality (scored 4–6 points)”, whereas no studies were considered to have “low quality (scored ≤3 points)”. The results of the appraisal are also summarized in Table S4. Two of the enrolled studies declared their conflict of interest with either an institution or company and two studies did not mention their funding source or conflict of interest.

### 3.3. Pooled Effects of the Primary Outcome

**Figure 2:**
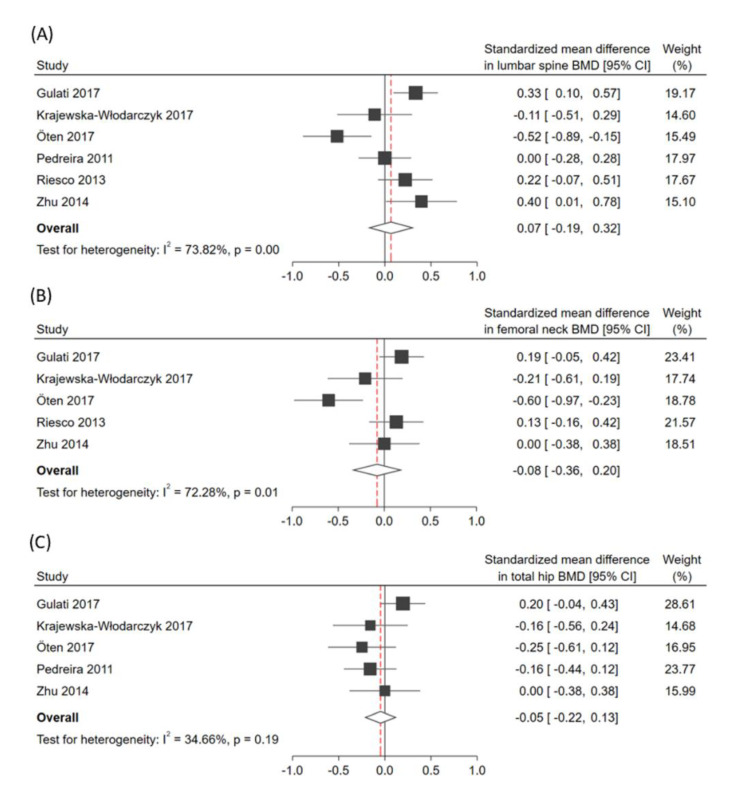
Forest plots of the standardized mean difference (SMD) in absolute bone mineral density. The plots are presented in subgroups of (**A**) lumbar spine, (**B**) femoral neck, and (**C**) total hip. CI, confidence interval.

In terms of the overall effect regarding the absolute BMD value, patients with psoriatic disease demonstrated no significantly decreased SMD despite different sites of measurement (SMD in lumbar spine: 0.07; 95% CI: −0.19 to 0.32; I^2^: 73.8%; SMD at femoral neck: −0.08; 95% CI: −0.36 to 0.20; I^2^: 72.3%; SMD at total hip: −0.05; 95% CI: −0.22 to 0.13; I^2^: 34.7%; Figure 2). Subgroup analysis in Table 1 revealed that the age of patients might be a moderator in lumbar spine BMD. After omitting the papers individually for sensitivity analysis, SMD results were similar to the above.

### 3.4. Pooled Effects of Secondary Outcomes

**Figure 3:**
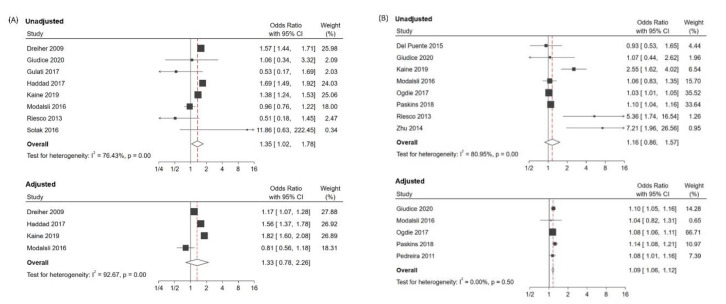
Forest plots of the unadjusted and adjusted odds ratio (OR) of osteoporosis (**A**) and fractures (**B**). CI, confidence interval.

As presented in Figure 3A, psoriatic patients tended to have a higher risk of developing osteoporosis before adjusting confounding factors (unadjusted OR: 1.35; 95% CI: 1.02–1.78, I^2^: 76.4%). However, after adjustment, patients with psoriatic disease were not likely to possess high ORs of developing osteoporosis (adjusted OR: 1.33; 95% CI: 0.78–2.26; I^2^: 92.7%) compared with the non-psoriatic controls. In Figure 3B, psoriatic patients were not likely to develop fractures before confounding adjustment (unadjusted OR: 1.16; 95% CI: 0.86–1.57; I^2^: 81.0%), but after adjusting for confounding factors, they possessed higher ORs of developing fractures compared with the non-psoriatic controls (adjusted OR: 1.09; 95% CI: 1.06–1.12; I^2^: 0%).

We focused on the adjusted estimates for drawing conclusions and compared them to the unadjusted estimates. The results were opposite before and after confounding adjustment, indicating the substantial role of confounding bias in terms of the overall effect size. Sensitivity analysis yielded similar results, making our pooled effects robust.

### 3.5. Heterogeneity and Small-Study Effects

Substantial heterogeneity was indicated in nearly all outcomes, except for the group regarding total hip BMD and the adjusted OR of fracture. Subgroup analyses revealed that psoriatic patients’ age might serve as a possible moderator in the primary outcome.

Small-study effects were detected using the Doi plot and LFK index. Major asymmetry was indicated in the subgroups of the femoral neck (LFK index: −2.60; Figure S1) in terms of the absolute value of BMD. Minor asymmetry was seen in the lumbar spine group regarding BMD (LFK index: −1.76; Figure S2) and in the adjusted outcome of osteoporosis (LFK index: 1.59; Figure S3). On the other hand, no asymmetry in the Doi plot was observed in the total hip group regarding BMD (LFK index: 0.60) and in the adjusted outcome of fracture (LFK index: −0.13), respectively.4. Discussion

Despite numerous studies concerning bone involvement in the investigative field of psoriatic disease, the findings remain controversial. In this systematic review and meta-analysis, we demonstrated no significant association between psoriatic disease and the absolute value of BMD in the lumbar spine, femoral neck, or total hip. Additionally, patients with psoriatic disease did not have higher risks of developing osteoporosis than the controls; nevertheless, they did have increased OR of sustaining fractures.

A fragility fracture is defined as a pathological fracture that results from low energy insults [51]. It is believed that fractures are associated with decreased bone strength, which reflects the integration of both bone quality and bone density [52]. However, the modalities measuring areal BMD (i.e., DXA) have limited ability to determine bone strength since they have limitations in measuring bone quality, such as microarchitecture, mineralization, collagen cross-links, crystal size, and marrow composition [53,54]. Therefore, osteoporosis defined by DXA may not reflect the actual bone strength reduction and may not serve as an accurate predictor for fractures. This inference may explain why psoriatic patients had increased fracture risk but displayed no association in BMD and osteoporosis in our meta-analysis.

Another explanation for our results is that the increased fracture risk may be attributed to reduced bone quality, namely, the depletion in bone microarchitecture and demineralization. Simon et al. [55] reported that the cortical and trabecular volumetric BMD was significantly decreased in the psoriatic population. Pfeil et al. [56] demonstrated periarticular demineralization in psoriatic disease by measuring the Metacarpal Index at the metacarpal bones. Further in vivo or in vitro experiments and clinical observation are required to clarify the pathogenic process.

Since the pathogenesis of psoriatic disease is complex and multifactorial, potential moderators were identified. Our subgroup analysis found that age may be a potential moderator for the analysis of BMD. Based on previous studies, aging may be related to bone loss with complex interaction between genetic, hormonal, biochemical, and environmental factors [57]. According to the World Health Organization, psoriasis most affects people at the age of 50–69 years [58]. In our study, the lumbar spine BMD increased in this age group, whereas it decreased in patients aged less than 50 years.

Apart from age, several possible confounding factors may affect our results. Previous studies suggested that low bone density in psoriatic disease was identified exclusively in men, usually less affected by bone destruction [16,17]. In contrast, one study reported that there was an increased BMD with postmenopausal women [59]. Hence, the sex of the patients may be a potential confounding factor. Additionally, chronic use of drugs that affect bone formation may also act as a confounding factor in our analysis. Systemic corticosteroids, methotrexate, and anti-TNF-α agents were reported to be either osteoporotic or anti-osteoporotic [27,28,29]. Finally, the body mass index of patients can also be considered a confounding factor. Epidemiologic research has indicated positive associations between obesity and bone health [60]; while adiposity and weight gain are associated with higher psoriasis risks [61].

Methodological problems regarding the representation of unpublished studies may have a considerable impact on the decision-making in clinical practice [62]. Concerns have been raised about the Egger’s asymmetry test and its power to detect asymmetry when the number of studies is small. We then used Doi plot and LFK index to evaluate small-study effects. Compared with the Egger’s *p* test, the LFK index had a superior area under the receiver operating characteristic curve (0.58 to 0.75 vs. 0.74 to 0.88, respectively) as well as higher sensitivity (18.5% to 43.0% vs. 71.3% to 72.1%, respectively) [39]. In contrast, the specificity is higher with the Egger’s *p* test (87.6% to 90.0% vs. 64.7–87.1%, respectively).

To the best of our knowledge, this is the first meta-analysis of observational studies to evaluate the BMD, osteoporosis, and fracture risk in adult patients with psoriatic disease (psoriasis and PsA). This analysis included not only osteoporosis and fracture risks, but also BMD measurements at different sites. This allowed us to estimate the total effect size with large sample size and a higher statistical power. Furthermore, we performed an up-to-date literature search and enrolled in the latest studies in the analysis. We applied sensitivity analyses after omitting each study one at a time, and the pooled results were robust with few changes. We used the novel Doi plot and LFK index to detect small-study effects.

There were some limitations in our studies. First, the inconsistency due to high between-study heterogeneity was observed. We were not able to perform meta-regression due to the availability of <10 studies in each outcome. Although it was a time-consuming effort, we used subgroup analysis to identify moderators in observational studies. Second, our study results could only explain the relationship between psoriatic disease and BMD, osteoporosis, and fractures. Further studies regarding pathogenetic clarification may be necessary. Third, while the LFK index has been demonstrated to discriminate asymmetry better and has higher sensitivity than the Egger’s *p*-value, its specificity is lower than that of the latter. Finally, the opposite results in the secondary outcomes revealed a crucial issue in terms of confounding factors in our enrolled studies, which can introduce bias; therefore, our results should be interpreted with caution.

## 4. Conclusions

Our results indicate that patients with psoriatic disease may be more likely to develop fractures compared with non-psoriatic controls. This higher risk for fracture may not necessarily associated with lower BMD nor a higher risk of osteoporosis. Future studies are warranted to establish stronger evidence regarding the understanding of bone strength and bone quality in patients with psoriasis or PsA. Based on our findings, we suggest that preventive measures for fractures may be beneficial in current clinical practice for such patients.

## Figures and Tables

**Table 1:** Subgroup analysis of primary outcome.

	Lumbar Spine			Femoral Neck			Total Hip		
Subgroups	N	SMD (95%CI)	I^2^ (%)	N	SMD (95%CI)	I^2^ (%)	N	SMD (95%CI)	I^2^ (%)
Study population									
Psoriasis	1	−0.07 (−0.40 to 0.27)	NA	0	NA	NA	1	−0.24 (−0.58 to 0.09)	NA
Psoriatic arthritis	6	0.07 (−0.19 to 0.33)	73.5	5	−0.08 (−0.36 to 0.20)	72.3	5	−0.04 (−0.22 to 0.14)	31.0
Study design									
Cross-sectional	5	0.00 (−0.28 to 0.29)	71.9	4	−0.16 (−0.49 to 0.17)	70.0	4	−0.15 (−0.32 to 0.03)	0
Cohort	1	0.33 (0.10 to 0.57) *	NA	1	0.19 (−0.05 to 0.42)	NA	1	0.20 (−0.04 to 0.43)	NA
Geographic location									
America	1	0.00 (−0.28 to 0.28)	NA	0	NA	NA	1	−0.16 (−0.44 to 0.12)	NA
Asia	2	−0.06 (−0.96 to 0.83)	91.3	2	−0.30 (−0.90 to 0.29)	80.1	2	−0.13 (−0.39 to 0.13)	0
Europe	3	0.19 (−0.04 to 0.42)	42.8	3	0.08 (−0.12 to 0.29)	29.5	2	0.06 (−0.28 to 0.40)	54.9
Age (years)									
<50	1	−0.52 (−0.89 to −0.15) *	NA	1	−0.60 (−0.97 to −0.23) *	NA	1	−0.25 (−0.61 to 0.12)	NA
50–59	3	0.31 (0.14 to 0.47) *	0	3	0.13 (−0.03 to 0.30)	0	2	0.14 (−0.06 to 0.34)	0
≥60	2	−0.04 (−0.27 to 0.19)	0	1	−0.21 (−0.61 to 0.19)	NA	2	−0.16 (−0.39 to 0.07)	0
BMI status									
Overweight	4	0.23 (0.06 to 0.40) *	26.2	3	0.13 (−0.03 to 0.30)	0	3	0.02 (−0.20 to 0.25)	45.4
Obesity	2	−0.32 (−0.72 to 0.08)	53.6	2	−0.41 (−0.80 to −0.03) *	50.6	2	−0.21 (−0.48 to 0.06)	0
Disease duration									
≥10 years	5	0.00 (−0.28 to 0.29)	71.9	4	−0.16 (−0.49 to 0.17)	70.0	4	−0.15 (−0.32 to 0.03)	0
<10 years	1	0.33 (0.10 to 0.57) *	NA	1	0.19 (−0.05 to 0.42)	NA	1	0.20 (−0.04 to 0.43)	NA
Medication use									
Yes	5	0.10 (−0.19 to 0.38)	77.6	4	−0.05 (−0.38 to 0.28)	77.7	4	−0.03 (−0.24 to 0.18)	47.0
No	1	−0.11 (−0.51 to 0.29)	NA	1	−0.21 (−0.61 to 0.19)	NA	1	−0.16 (−0.56 to 0.24)	NA
Risk of bias									
NOS ≥ 7	2	0.29 (0.11 to 0.47) *	0	2	0.16 (−0.02 to 0.35)	0	1	0.20 (−0.04 to 0.43)	NA
NOS < 7	4	−0.06 (−0.41 to 0.29)	74.3	3	−0.27 (−0.63 to 0.08)	61.4	4	−0.15 (−0.32 to 0.03)	0

* *p* < 0.05 N, Number of studies; SMD, standardized mean difference; CI, confidence interval; NA, not applicable; BMI, body mass index; DXA, dual-energy X-ray absorptiometry; NOS, Newcastle–Ottawa Scale.

## Supplementary Materials

The following are available online at https://www.mdpi.com/2077-0383/9/11/3712/s1, Methods: Detailed search strategy modified to accommodate different databases; Table S1: Modified Newcastle–Ottawa Scale for cohort studies; Table S2: Modified Newcastle-Ottawa Scale for case-control studies; Table S3: Modified Newcastle-Ottawa Scale for cross-sectional studies; Table S4: Characteristics of included studies; Figure S1: Doi plot and LFK index of femoral neck BMD; Figure S2: Doi plot and LFK index of lumbar spine BMD; Figure S3: Doi plot and LFK index of osteoporosis.

## References

[1] Armstrong A.W., Read C. (2020). Pathophysiology, Clinical Presentation, and Treatment of Psoriasis. JAMA.

[2] Ritchlin C.T., Colbert R.A., Gladman D.D. (2017). Psoriatic Arthritis. N. Engl. J. Med..

[3] Bilal J., Malik S.U., Riaz I.B., Kurtzman D.J. (2018). Psoriasis and Psoriatic Spectrum Disease: A Primer for the Primary Care Physician. Am. J. Med..

[4] Li Q., Chandran V., Tsoi L., O’Rielly D., Nair R.P., Gladman D., Elder J.T., Rahman P. (2020). Quantifying Differences in Heritability among Psoriatic Arthritis (PsA), Cutaneous Psoriasis (PsC) and Psoriasis vulgaris (PsV). Sci. Rep..

[5] Stuart P.E., Nair R.P., Tsoi L.C., Tejasvi T., Das S., Kang H.M., Ellinghaus E., Chandran V., Callis-Duffin K., Ike R. (2015). Genome-wide Association Analysis of Psoriatic Arthritis and Cutaneous Psoriasis Reveals Differences in Their Genetic Architecture. Am. J. Hum. Genet..

[6] Sakkas L.I., Bogdanos D.P. (2017). Are psoriasis and psoriatic arthritis the same disease? The IL-23/IL-17 axis data. Autoimmun. Rev..

[7] Blauvelt A., Chiricozzi A. (2018). The Immunologic Role of IL-17 in Psoriasis and Psoriatic Arthritis Pathogenesis. Clin. Rev. Allergy Immunol..

[8] Okhovat J.-P., Ogdie A., Reddy S., Rosen C.F., Scher J.U., Merola J.F. (2017). Psoriasis and Psoriatic Arthritis Clinics Multicenter Advancement Network Consortium (PPACMAN) Survey: Benefits and Challenges of Combined Rheumatology-dermatology Clinics. J. Rheumatol..

[9] Savage L., Tinazzi I., Zabotti A., Laws P.M., Wittmann M., McGonagle D. (2020). Defining Pre-Clinical Psoriatic Arthritis in an Integrated Dermato-Rheumatology Environment. J. Clin. Med..

[10] Alivernini S., Tolusso B., Petricca L., Bui L., Di Sante G., Peluso G., Benvenuto R., Fedele A.L., Federico F., Ferraccioli G. (2017). Synovial features of patients with rheumatoid arthritis and psoriatic arthritis in clinical and ultrasound remission differ under anti-TNF therapy: A clue to interpret different chances of relapse after clinical remission?. Ann. Rheum. Dis..

[11] Sirufo M.M., De Pietro F., Bassino E.M., Ginaldi L., De Martinis M. (2020). Osteoporosis in Skin Diseases. Int. J. Mol. Sci..

[12] Paine A., Ritchlin C. (2018). Altered Bone Remodeling in Psoriatic Disease: New Insights and Future Directions. Calcif. Tissue Int..

[13] Elmets C.A., Leonardi C.L., Davis D.M., Gelfand J.M., Lichten J., Mehta N.N., Armstrong A.W., Connor C., Cordoro K.M., Elewski B.E. (2019). Joint AAD-NPF guidelines of care for the management and treatment of psoriasis with awareness and attention to comorbidities. J. Am. Acad. Dermatol..

[14] Oliveira M.D.F.S.P.D., Rocha B.D.O., Duarte G.V. (2015). Psoriasis: Classical and emerging comorbidities. An. Bras. Dermatol..

[15] Chandran S., Aldei A., Johnson S.R., Cheung A.M., Salonen D., Gladman D.D. (2016). Prevalence and risk factors of low bone mineral density in psoriatic arthritis: A systematic review. Semin. Arthritis Rheum..

[16] Dreiher J., Weitzman D., Cohen A.D. (2009). Psoriasis and Osteoporosis: A Sex-Specific Association?. J. Investig. Dermatol..

[17] Lajevardi V., Abedini R., Moghaddasi M., Nassiri S., Goodarzi A. (2017). Bone mineral density is lower in male than female patients with plaque-type psoriasis in Iran. Int. J. Women’s Dermatol..

[18] Ogdie A., Harter L., Shin D., Baker J., Takeshita J., Choi H.K., Love T.J., Gelfand J.M. (2017). The risk of fracture among patients with psoriatic arthritis and psoriasis: A population-based study. Ann. Rheum. Dis..

[19] Paskins Z., Whittle R., Sultan A.A., Müller S., Blagojevic-Bucknall M., Helliwell T., Packham J., Hider S., Roddy E., Mallen C. (2018). Risk of fragility fracture among patients with late-onset psoriasis: A UK population-based study. Osteoporos. Int..

[20] Harrison B.J., Hutchinson C.E., Adams J., Bruce I.N., Herrick A.L. (2002). Assessing periarticular bone mineral density in patients with early psoriatic arthritis or rheumatoid arthritis. Ann. Rheum. Dis..

[21] Busquets N., Gómez-Vaquero C., Rodríguez-Rodríguez L., Vilaseca D.R., Narváez J., Carmona L., Nolla J.M. (2014). Bone mineral density status and frequency of osteoporosis and clinical fractures in 155 patients with psoriatic arthritis followed in a university hospital. Reumatol. Clin..

[22] Giudice L.F.L., Scolnik M., Pierini F.S., Zucaro N.M.M., Gallego J.F.J., Soriano E.R. (2020). Fragility fractures in psoriatic arthritis patients: A matched retrospective cohort study. Clin. Rheumatol..

[23] Modalsli E., Åsvold B., Romundstad P., Langhammer A., Hoff M., Forsmo S., Naldi L., Saunes M. (2017). Psoriasis, fracture risk and bone mineral density: The HUNT Study, Norway. Br. J. Dermatol..

[24] Paschalis E.P., Shane E., Lyritis G., Skarantavos G., Mendelsohn R., Boskey A.L. (2004). Bone Fragility and Collagen Cross-Links. J. Bone Miner. Res..

[25] Moher D., Liberati A., Tetzlaff J., Altman D.G., PRISMA Group (2009). Preferred reporting items for systematic reviews and meta-analyses: The PRISMA statement. BMJ.

[26] Stroup D.F., Berlin J.A., Morton S.C., Olkin I., Williamson G.D., Rennie D., Moher D., Becker B.J., Sipe T.A., Thacker S.B. (2000). Meta-analysis of Observational Studies in Epidemiology: A Proposal for Reporting. JAMA.

[27] Buckley L., Humphrey M.B. (2018). Glucocorticoid-Induced Osteoporosis. N. Engl. J. Med..

[28] Orsolini G., Fassio A., Rossini M., Adami G., Giollo A., Caimmi C., Idolazzi L., Viapiana O., Gatti D. (2019). Effects of biological and targeted synthetic DMARDs on bone loss in rheumatoid arthritis. Pharmacol. Res..

[29] Kawai V.K., Stein C.M., Perrien D.S., Griffin M.R. (2012). Effects of anti-tumor necrosis factor α agents on bone. Curr. Opin. Rheumatol..

[30] Wells G.A., Shea B., O’Connell D., Robertson J., Peterson J., Welch V., Losos M., Tugwell P. The Newcastle–Ottawa Scale (NOS) for Assessing the Quality of Nonrandomised Studies in Meta-Analyses. http://www.ohri.ca/programs/clinical_epidemiology/oxford.htm.

[31] Anglin R.E.S., Samaan Z., Walter S.D., McDonald S.D. (2013). Vitamin D deficiency and depression in adults: Systematic review and meta-analysis. Br. J. Psychiatry.

[32] Higgins J.P.T., Thomas J., Chandler J., Cumpston M., Li T., Page M.J., Welch V.A. (2020). Cochrane Handbook for Systematic Reviews of Interventions Version 6.1 [Updated September 2020].

[33] Dekkers O.M., Vandenbroucke J.P., Cevallos M., Renehan A.G., Altman D.G., Egger M. (2019). COSMOS-E: Guidance on conducting systematic reviews and meta-analyses of observational studies of etiology. PLoS Med..

[34] Metelli S., Chaimani A. (2020). Challenges in meta-analyses with observational studies. Evid. Based Ment. Health.

[35] Guolo A., Varin C. (2015). Random-effects meta-analysis: The number of studies matters. Stat. Methods Med. Res..

[36] Röver C., Knapp G., Friede T. (2015). Hartung-Knapp-Sidik-Jonkman approach and its modification for random-effects meta-analysis with few studies. BMC Med. Res. Methodol..

[37] Veroniki A.A., Jackson D., Bender R., Kuss O., Langan D., Higgins J.P., Knapp G., Salanti G. (2019). Methods to calculate uncertainty in the estimated overall effect size from a random-effects meta-analysis. Res. Synth. Methods.

[38] Higgins J.P.T., Thompson S.G., Deeks J.J., Altman D.G. (2003). Measuring inconsistency in meta-analyses. BMJ.

[39] Furuya-Kanamori L., Barendregt J.J., Doi S.A.R. (2018). A new improved graphical and quantitative method for detecting bias in meta-analysis. Int. J. Evid.-Based Heal..

[40] Del Puente A., Esposito A., Costa L., Benigno C., Foglia F., Oriente A., Bottiglieri P., Caso F., Scarpa R. (2015). Fragility Fractures in Patients with Psoriatic Arthritis. J. Rheumatol. Suppl..

[41] Gulati A.M., Hoff M., Salvesen Ø., Dhainaut A., Semb A.G., Kavanaugh A., Haugeberg G. (2017). Bone mineral density in patients with psoriatic arthritis: Data from the Nord-Trøndelag Health Study 3. RMD Open.

[42] Haddad A., Ashkenazi R.I., Bitterman H., Feldhamer I., Greenberg-Dotan S., Lavi I., Batat E., Bergman I., Cohen A.D., Zisman D. (2017). Endocrine Comorbidities in Patients with Psoriatic Arthritis: A Population-based Case-controlled Study. J. Rheumatol..

[43] Kaine J., Song X., Kim G., Hur P., Palmer J.B. (2019). Higher Incidence Rates of Comorbidities in Patients with Psoriatic Arthritis Compared with the General Population Using U.S. Administrative Claims Data. J. Manag. Care Spec. Pharm..

[44] Krajewska-Włodarczyk M., Owczarczyk-Saczonek A., Placek W. (2017). Changes in body composition and bone mineral density n postmenopausal women with psoriatic arthritis. Reumatologia.

[45] Oten E., Baskan B., Sivas F., Bodur H. (2017). Relation Between Osteoporosis and Vitamin D Levels and Disease Activity in Psoriatic Arthritis. Erciyes Med. J..

[46] Pedreira P.G., Pinheiro M.M., Szejnfeld V.L. (2011). Bone mineral density and body composition in postmenopausal women with psoriasis and psoriatic arthritis. Arthritis Res..

[47] Riesco M., Manzano F., Font P., García A., Nolla J.M. (2013). Osteoporosis in psoriatic arthritis: An assessment of densitometry and fragility fractures. Clin. Rheumatol..

[48] Solak B., Dikicier B.S., Celik H.D., Erdem T. (2016). Bone Mineral Density, 25-OH Vitamin D and Inflammation in Patients with Psoriasis. Photodermatol. Photoimmunol. Photomed..

[49] Zhu T.Y., Griffith J.F., Qin L., Hung V.W.Y., Fong T.-N., Au S.-K., Kwok A., Leung P.-C., Li E.K., Tam L. (2014). Density, structure, and strength of the distal radius in patients with psoriatic arthritis: The role of inflammation and cardiovascular risk factors. Osteoporos. Int..

[50] Weir C.B., Jan A. (2019). BMI Classification Percentile and Cut Off Points.

[51] Papaioannou A., Morin S., Cheung A.M., Atkinson S., Brown J.P., Feldman S., Hanley D.A., Hodsman A., Jamal S.A., Kaiser S.M. (2010). 2010 clinical practice guidelines for the diagnosis and management of osteoporosis in Canada: Summary. Can. Med. Assoc. J..

[52] NIH Consensus Development Panel on Osteoporosis Prevention, Diagnosis, and Therapy (2001). Osteoporosis Prevention, Diagnosis, and Therapy. JAMA.

[53] Ott S.M. (2016). Bone strength: More than just bone density. Kidney Int..

[54] Torres-Del-Pliego E., Vilaplana L., Güerri-Fernández R., Diez-Perez A. (2013). Measuring Bone Quality. Curr. Rheumatol. Rep..

[55] Simon D., Haschka J., Muschitz C., Kocijan A., Baierl A., Kleyer A., Schett G., Kapiotis S., Resch H., Sticherling M. (2020). Bone microstructure and volumetric bone mineral density in patients with hyperuricemia with and without psoriasis. Osteoporos. Int..

[56] Pfeil A., Krojniak L., Renz D.M., Reinhardt L., Franz M., Oelzner P., Wolf G., Böttcher J. (2016). Psoriatic arthritis is associated with bone loss of the metacarpals. Arthritis Res..

[57] Demontiero O., Vidal C., Duque G. (2012). Aging and bone loss: New insights for the clinician. Ther. Adv. Musculoskelet. Dis..

[58] Global Burden of Disease Collaborative Network (2012). Global Burden of Disease Study 2010 (GBD 2010) Results by Cause 1990–2010.

[59] Osmancevic A., Landin-Wilhelmsen K., Larkö O., Mellström D., Wennberg A.-M., Hulthén L., Krogstad A.-L. (2008). Risk factors for osteoporosis and bone status in postmenopausal women with psoriasis treated with UVB therapy. Acta. Derm. Venereol..

[60] Lee S.J., Lee J., Sung J. (2019). Obesity and Bone Health Revisited: A Mendelian Randomization Study for Koreans. J. Bone Miner. Res..

[61] Aune D., Snekvik I., Schlesinger S., Norat T., Riboli E., Vatten L.J. (2018). Body mass index, abdominal fatness, weight gain and the risk of psoriasis: A systematic review and dose–response meta-analysis of prospective studies. Eur. J. Epidemiol..

[62] Atakpo P., Vassar M. (2016). Publication bias in dermatology systematic reviews and meta-analyses. J. Dermatol. Sci..

