# Supplementary material for: Bone Mineral Density, Osteoporosis, and Fracture Risk in Adult Patients with Psoriasis or Psoriatic Arthritis: A Systematic Review and Meta-Analysis of Observational Studies
Source: J Clin Med. 2020 Nov 19;9(11):3712. doi: 10.3390/jcm9113712 (PMC7699147; doi:10.3390/jcm9113712)
Supplement: Supplementary file 1 [file jcm-09-03712-s001.pdf]

Supplemental Materials

Methods. Detailed search strategy modified to accommodate different databases
Table S1. Modified Newcastle-Ottawa Scale for cohort studies
Table S2. Modified Newcastle-Ottawa Scale for case-control studies
Table S3. Modified Newcastle-Ottawa Scale for cross-sectional studies
Table S4. Characteristics of included studies
Figure S1. Doi plot and LFK index of femoral neck BMD
Figure S2. Doi plot and LFK index of lumbar spine BMD
Figure S3. Doi plot and LFK index of osteoporosis

Methods. Detailed search strategy modified to accommodate different databases

PubMed

1. Psoriasis OR Psoriases OR Pustulosis of Palms and Soles OR Pustulosis Palmaris et Plantaris OR Palmoplantaris Pustulosis OR Pustular Psoriasis of Palms and Soles
2. Psoriatic Arthritis OR Arthritic Psoriasis OR Psoriasis Arthropathica OR Psoriatic Arthropathy OR Psoriatic Arthropathies
3. 1 OR 2
4. Bone Density OR Bone Mineral Density OR Bone Mineral Densities OR Bone Mineral Content OR Bone Mineral Contents
5. Osteoporosis OR Osteoporoses OR Bone Loss OR Bone Losses
6. Fracture OR Fractures OR Broken Bone OR Broken Bones
7. 4 OR 5 OR 6

8. 3 AND 7

Embase

1. exp PSORIASIS/
2. psoria$.mp.
3. palmoplantar$ pustulosis.mp.
4. pustulosis palmaris et plantaris.mp.
5. (pustulosis and palms and soles).mp.
6. 1 or 2 or 3 or 4 or 5
7. exp PSORIATIC ARTHRITIS/
8. psoriatic arthr*.mp.
9. arthritic psorias?s.mp.
10. 7 or 8 or 9
11. 6 or 10
12. (bone density or BMD).ti,ab. or Bone Density/
13. bone mineral density.ti,ab.
14. (bone mineral content or BMC).ti,ab.
15. (bone adj (mass or strength or loss or accret$ or remodel$ or resorp$)).ti,ab.
16. 12 or 13 or 14 or 15
17. osteoporo$.ti,ab. or Osteoporosis/
18. bone loss$.ti,ab. or Bone Loss$/
19. 17 or 18
20. fracture$.ti,ab. or Fractures, Bone/
21. broken bone$.ti,ab. or Broken Bone$/
22. 20 or 21
23. 16 or 19 or 22
24. 11 and 23

Cochrane Library

1. Psoriasis or Psoriases or Pustulosis of Palms and Soles or Pustulosis Palmaris et Plantaris or Palmoplantaris Pustulosis or Pustular Psoriasis of Palms and Soles
2. Psoriatic Arthritis or Arthritic Psoriasis or Psoriasis Arthropathica or Psoriatic Arthropathy or Psoriatic Arthropathies
3. #1 or #2
4. Bone Density or Bone Mineral Density or Bone Mineral Densities or Bone Mineral Content or Bone Mineral Contents
5. Osteoporosis or Osteoporoses or Bone Loss or Bone Losses
6. Fracture or Fractures or Broken Bone or Broken Bones
7. 4 or 5 or 6
8. 3 and 7

Web of science

1. ALL=(Psorias?s OR Pustul* of Palms and Soles OR Pustulosis Palmaris et Plantaris OR Palmoplantar* Pustulosis OR (Pustular Psorias?s AND Palms AND Soles))
2. ALL=(Psoriatic Arthr* OR Arthritic Psorias?s OR Psoriasis Arthropathica)
3. 1 OR 2
4. ALL=(Bone Density OR Bone Mineral Densit* OR Bone Mineral Content*)
5. ALL=(Osteoporos?s OR Bone Loss*)
6. ALL=(Fracture* OR Broken Bone*)
7. 4 OR 5 OR 6
8. 3 AND 7

Airiti Library

[ALL]:psoriasis AND [ALL]:"bone mineral density"

Chinese National Knowledge Infrastructure

((Psoriasis) OR (Psoriatic arthritis)) AND ((Bone mineral density) OR (Osteoporosis) OR (Fracture))

Summary of database search

PubMed: 606
Embase: 2,014
Cochrane Library: 112
Web of Science: 267

Airiti Library: 153
Chinese National Knowledge Infrastructure: 148

Table S1. Modified Newcastle-Ottawa Scale for cohort studies (maximum: 9 stars)

**Selection**
1) Representativeness of the exposed cohort (psoriatic disease)<br>a) truly or somewhat representative of the general population of psoriatic disease *<br>b) potential for selection biases or not stated<br>c) no description
2) Selection of non-psoriatic controls<br>a) drawn from the same community as the exposed cohort *<br>b) drawn from a different source<br>c) no description
3) Ascertainment of psoriatic disease<br>a) secure records (hospital records based on clinical or histological information) *<br>b) International Classification of Diseases (ICD) code*<br>c) self-reports<br>d) no description
4) Demonstration that outcome of interest was not present at start of study<br>a) yes *<br>b) no
**Comparability (maximum: 2 stars)**
1) Comparability of the exposed group and the non-psoriatic controls on the basis of the design or analysis<br>a) study controls for age*<br>b) study controls for any additional factor *<br>c) no description
**Outcome**
1) Assessment of outcome<br>a) secure record (records of bone mineral density measurement, osteoporosis, or fracture) *<br>b) structured interview *<br>c) written self-report only<br>d) no description
2) Was follow-up long enough for outcome<br>a) yes (≥ 5 years) *<br>b) no (< 5 years)
3) Adequacy of follow up of cohorts<br>a) complete follow up-all subjects accounted *<br>b) subjects lost to follow up unlikely to introduce bias (loss of follow-up rate <20%) *<br>c) loss of follow-up rate >80%<br>d) no description of the lost

Table S2. Modified Newcastle-Ottawa Scale for case-control studies (maximum: 9 stars)

**Selection**
1) Representativeness of the cases (psoriatic disease)<br>a) truly or somewhat representative of the general population of psoriatic disease *<br>b) potential for selection biases or not stated<br>c) no description
2) Selection of non-psoriatic controls<br>a) drawn from the same community as the exposed cohort *<br>b) drawn from a different source<br>c) no description
3) Ascertainment of psoriatic disease<br>a) secure records (hospital records based on clinical or histological information) *<br>b) International Classification of Diseases (ICD) code *<br>c) self-reports<br>d) no description
4) Demonstration that outcome of interest was not present at start of study<br>a) yes *<br>b) no
**Comparability (maximum: 2 stars)**
1) Comparability of the exposed group and the non-psoriatic controls on the basis of the design or analysis<br>a) study controls for age *<br>b) study controls for any additional factor *<br>c) no description
**Outcome**
1) Assessment of outcome<br>a) secure record (records of bone mineral density measurement, osteoporosis, or fracture) *<br>b) structured interview *<br>c) written self-report only<br>d) no description
2) Same method of ascertainment for cases and outcomes<br>a) yes *<br>b) no
3) Non-response rate<br>a) same rate for both groups *<br>b) non respondents described<br>c) no description

Table S3. Modified Newcastle-Ottawa Scale for cross-sectional studies (maximum: 6 stars)

**Selection**
1) Representativeness of the exposed group (psoriatic disease)<br>a) truly or somewhat representative of the general population of psoriatic disease *<br>b) potential for selection biases or not stated
2) Selection of non-psoriatic controls<br>a) drawn from the same community as the exposed group *<br>b) drawn from a different source<br>c) no description
3) Ascertainment of psoriatic disease<br>a) secure records (hospital records based on clinical or histological information) *<br>b) International Classification of Diseases (ICD) code*<br>c) self-reports<br>d) no description
**Comparability**
1) Comparability of the exposed group and the non-psoriatic controls on the basis of the design or analysis<br>a) study controls for age *<br>b) study controls for any additional factor *<br>c) no description
**Outcome**
1) Assessment of outcome<br>a) secure record (records of bone mineral density measurement, osteoporosis, or fracture) *<br>b) structured interview *<br>c) written self-report only<br>d) no description

Table 4. Characteristics of included studies.

First author, year	Study design	Country	No. of participants	Age (years) mean ± SD or range	Sex (female %)	BMI (kg/m^2) mean ± SD	Disease duration (years) mean ± SD	Medication[@] use	BMD measurement			Outcomes regarding osteoporosis or fracture (adjusted covariates)	Quality NOS[$]	COI
									Device[#]	site	outcome			
Del Puente, 2015	case-control	Italy	PsA: 92 Control: 100	62.2 ± 7.3	100	24.1 ± 3.7	7.9 ± 4.5	yes	DXA	FN, TH	NA	prevalence of fracture	S*** C** O*	NA
Dreiher, 2009	case-control	Israel	Pso: 7,936 Control: 14,835	65.6 ± 10.1	48.2	NA	NA	yes	NA	NA	NA	adjusted OR for osteoporosis (adjusted for age and comorbidities)	S**** C** O**	none
Giudice, 2020	retrospective cohort	Argentina	PsA: 92 Control: 184	50.2 ± 16.2	47.8	NA	NA	yes	DXA	NA	NA	prevalence of osteoporosis and fracture, adjusted HR for fracture (adjusted for age)	S**** C** O***	none
Gulati, 2017	prospective cohort	Norway	PsA: 69 Control: 11,703	56.8 ± 12.5	65.2	28.5 ± 4.3	8.3 ± 6.8	yes	DXA (GE lunar)	LS, FN, TH	absolute BMD, T score	prevalence of osteoporosis	S**** C** O***	none
Haddad, 2017	case-control	Israel	PsA: 3,161 Control: 31,610	58.4 ± 15.4	53.4	27.5 ± 5.8	NA	yes	NA	NA	NA	unadjusted and adjusted OR for osteoporosis (adjusted for age, sex, smoking, and steroid use)	S**** C** O**	NA
Kaine, 2019	retrospective cohort	USA	PsA: 14,898 Control: 35,037	53.4 ± 12.4	55.4	NA	NA	NA	NA	NA	NA	prevalence of osteoporosis and fracture, adjusted HR for osteoporosis (adjusted for age, gender, region, health plan, urbanicity, index year, DCCI, and comorbidities)	S**** C** O***	yes
Krajewska-Włodarczyk, 2017	cross-sectional	Poland	PsA: 51 Control: 44	65.6 ± 5.9	100	30.1 ± 5.8	11.1 ± 8.9	no	DXA	LS, FN, TH	absolute BMD, T score, Z score	NA	S** C O*	none
Modalsli, 2016	cross-sectional for osteoporosis; prospective cohort for fracture	Norway	Pso: 2,804 Control: 45,390	≥ 20	52	25-30	NA	yes	DXA (GE lunar)	NA	mean difference in T score	prevalence of osteoporosis and fracture, adjusted OR for osteoporosis, adjusted HR for fracture (adjusted for age, sex, BMI, education, smoking, and systemic steroid use)	S*** C** O***	none
Ogdie, 2017	retrospective cohort	UK	Pso: 158,323 PsA: 9,788 Control: 821,834	46.8 ± 17.3	53.3	26.8 ± 5.6	NA	yes	NA	NA	NA	unadjusted and adjusted HR of fracture (adjusted for age, sex, BMI, smoking, comorbidities, and medication)	S**** C** O***	yes
Öten, 2017	cross-sectional	Turkey	PsA: 58 Control: 58	42.6 ± 8.4	56.9	30.3 ± 5.7	16.0 ± 10.5	yes	DXA (Hologic)	LS, FN, TH	absolute BMD	NA	S* C O*	none
Paskins, 2018	retrospective cohort	UK	Pso: 24,219 Control: 94,820	58.9 ± 11.8	49.1	25-30	NA	yes	NA	NA	NA	prevalence, unadjusted HR, and adjusted HR of fracture (adjusted for age, sex, BMI,	S**** C** O***	none

												alcohol consumption, smoking, Charlson comorbidity, and medication)		
Pedreira, 2011	cross-sectional	Brazil	Pso: 52<br>PsA: 45<br>Control: 98	61.0 ± 8.9	100	27.8 ± 5.1	23.2 ± 17.0	yes	DXA (GE lunar)	LS, TH	absolute BMD	adjusted OR for fracture (adjusted for age and weight)	S**<br>C**<br>O*	none
Riesco, 2013	cross-sectional	Spain	PsA: 91<br>Control: 91	54.0 ± 12.8	50.5	28.6 ± 4.3	12.7 ± 8.3	yes	DXA (Norland)	LS, FN	absolute BMD, T score, Z score	prevalence of osteoporosis and fracture	S***<br>C**<br>O*	none
Solak, 2016	case-control	Turkey	Pso: 43<br>Control: 41	35.3 ± 8.7	62.8	24.9 ± 3.6	<10	yes	DXA (Hologic)	LS, FN, TH	T score, Z score	prevalence of osteoporosis	S**<br>C*<br>O**	none
Zhu, 2014	cross-sectional	China	PsA: 53<br>Control: 53	53.1 ± 8.9	54.7	25.2 ± 3.7	14.0 ± 7.2	yes	DXA (Hologic)	LS, FN, TH	absolute BMD	prevalence of fracture	S**<br>C<br>O*	none

@ We demonstrated potential osteoporotic/anti-osteoporotic drug usage, such as systemic corticosteroid, methotrexate, or anti-TNF alpha biologic agents.

[#] DXA manufacturer was listed if available.

$ Newcastle-Ottawa Scale was scored with stars. (S, selection of study groups; C, comparability of study groups; O, ascertainment of outcome of interest)

No. number; SD, standard deviation; BMI, body mass index; BMD, bone mineral density; NOS: Newcastle-Ottawa Scale; COI, conflict of interest; Pso: psoriasis; PsA: psoriatic arthritis; NA, not applicable; DXA, dual energy x-ray absorptiometry; LS, lumbar spine; FN, femoral neck, TH, total hip; OR, odds ratio; HR, hazard ratio; DCCI, Deyo Charlson Comorbidity Index; USA, United States of America; UK, United Kingdom

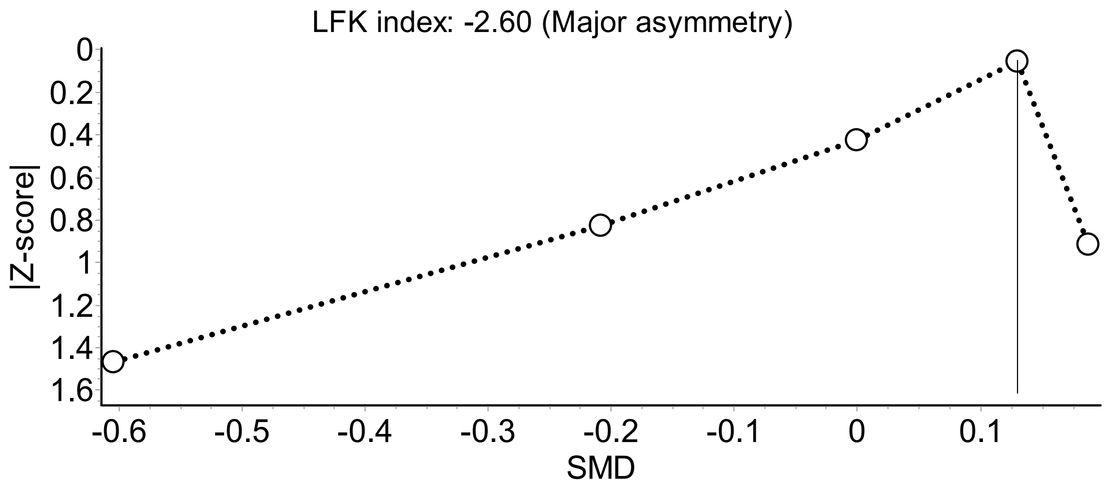

Figure 1. Doi plot and LFK index of femoral neck BMD.

LFK index: Luis Furuya–Kanamori index; SMD, standardized mean difference.

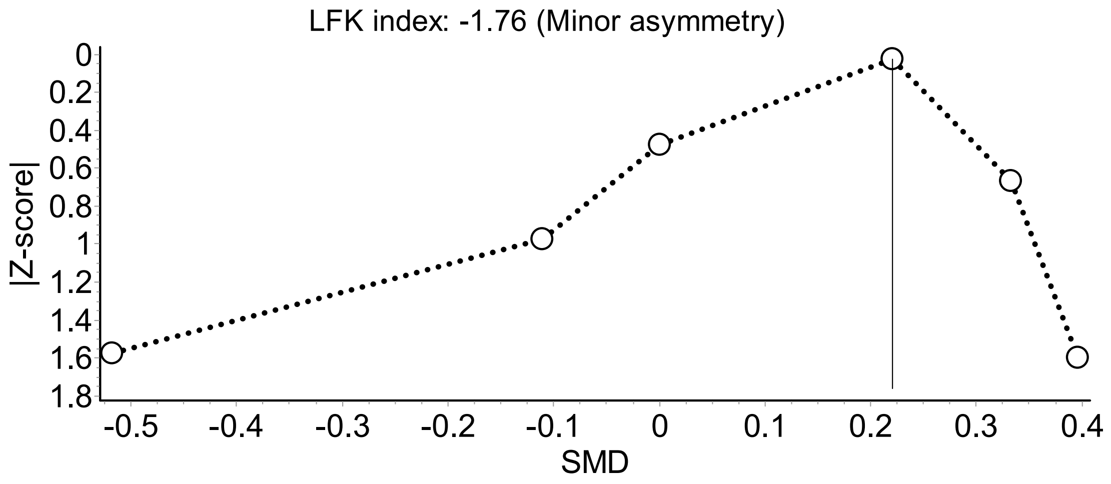

Figure 2. Doi plot and LFK index of lumbar spine BMD.

LFK index: Luis Furuya–Kanamori index; SMD, standardized mean difference.

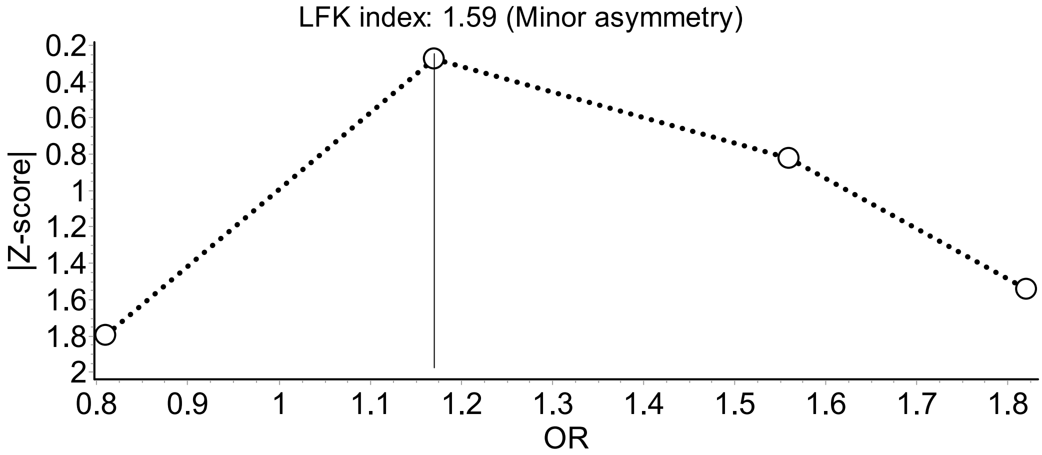

Figure 3. Doi plot and LFK index of osteoporosis.

LFK index: Luis Furuya–Kanamori index; OR, odds ratio.
